# Occult HBV infection

**DOI:** 10.1007/s00281-012-0327-7

**Published:** 2012-07-26

**Authors:** Giovanni Raimondo, Gaia Caccamo, Roberto Filomia, Teresa Pollicino

**Affiliations:** Unit of Clinical and Molecular Hepatology, Department of Internal Medicine, University Hospital of Messina, Messina, Italy

**Keywords:** Hepatitis B virus (HBV), Occult HBV infection (OBI), HBV replication and gene expression, HBV cccDNA, HBV transmission, HBV-related liver disease, HBV oncogenecity

## Abstract

The long-lasting persistence of hepatitis B virus (HBV) genomes in the liver (with detectable or undetectable HBV DNA in the serum) of individuals testing negative for the HBV surface antigen (HBsAg) is termed occult HBV infection (OBI). Although in a minority of cases the lack of HBsAg detection is due to infection with variant viruses unrecognized by available assays (S-escape mutants), the typical OBI is related to replication-competent HBVs strongly suppressed in their replication activity. The causes of HBV suppression are not yet well clarified, although the host’s immune surveillance and epigenetic mechanisms are likely involved. OBI is a worldwide diffused entity, but the available data of prevalence in various categories of individuals are often contrasting because of the different sensitivity and specificity of the methods used for its detection in many studies. OBI may have an impact in several different clinical contexts. In fact, it can be transmitted (i.e., through blood transfusion and liver transplantation) causing classic forms of hepatitis B in newly infected individuals. The development of an immunosuppressive status (mainly by immunotherapy or chemotherapy) may induce OBI reactivation and development of acute and often severe hepatitis. Finally, evidence suggests that OBI can favor the progression of liver fibrosis, in particular in HCV-infected patients. The possible contribution of OBI to the establishment of cirrhosis also implies its possible indirect role in the development of hepatocellular carcinoma. On the other hand, OBI may maintain most of the direct transforming properties of the overt HBV infection, such as the capacity to integrate in the host’s genome and to synthesize pro-oncogenic proteins.

According to European guidelines on management of chronic hepatitis B (CHB) virus (HBV) infection, the natural history of CHB can be schematically divided into five—not necessarily sequential and stable—phases [1], the fifth of which is occult HBV infection (OBI) that a panel of experts recently defined as the presence of viral DNA in the liver (with detectable or undetectable HBV DNA in the serum) of individuals testing negative for the HBV surface antigen (HBsAg) [2]. Suspected to exist since the 70s and the occasional topic of a few important studies for more than two decades, general interest in OBI arose and became a major issue of hepatology research from 1999 when The New England Journal of Medicine published a study performed by testing the liver biopsy specimens from a large series of HBsAg-negative patients with chronic liver disease (CLD) for HBV genomes [3]. In fact, this study provided new insight in both the virological aspects and the possible clinical implications of OBI showing that (a) it may favor or accelerate the progression toward cirrhosis of hepatitis C virus (HCV)-related chronic hepatitis and that (b) “occult” viruses usually have no genetic mutations capable of preventing viral replication as well as HBsAg synthesis (Table 1). As a consequence of the growing interest in OBI that followed the publication of this paper [2–15], we are observing a considerable, continuous increase of the number of studies in this field published by journals covering different areas of biomedical interest (Fig. 1). In this review, we aimed at revising the collection of data on OBI, also stressing the aspects that are largely accepted by the scientific community and those that are still debated.

**Table 1:** Milestones in the progression of knowledge of occult HBV infection

Year—journal—authors	Topic
1975—Gastroenterology—Wands et al. [4]	OBI reactivation in patients undergoing chemotherapy
1978—N Engl J Med—Hoofnagle et al. [13]	HBV transmission by blood transfusion from an OBI donor
1981—N Engl J Med—Shafritz et al. [7]	HBV DNA integration in the hepatocyte genome of HBsAg-negative individuals
1981—Proc Natl Acad Sci—Brechot et al. [15]	
1988—Lancet—Thiers et al. [6]	Acute hepatitis B in chimpanzees injected with HBV isolates from blood of OBI carriers
1989—Proc Natl Acad Sci—Kaneko et al. [12]	Polymerase chain reaction detection of HBV DNA in serum of HBsAg-negative individuals
1994—Lancet—Chazouillères et al. [14]	Liver transplant from OBI donors may induce hepatitis B in recipients
1994—J Clin Invest—Michalak et al. [11]	OBI in patients recovered from acute hepatitis B
1996—Nature Medicine—Rehermann et al. [8]	A strong CTL-specific anti-HBV response persists over time in patients who recovered from acute hepatitis B
1996—J Clin Invest—Penna et al. [10]	
1999—N Engl J Med—Cacciola et al. [3]	OBI is associated with cirrhosis in patients with chronic hepatitis C and the virus is wild-type
2002—Lancet Inf Dis—Torbenson and Thomas [5]	First systematic review of the OBI field
2004—Gastroenterology—Pollicino et al. [9]	Molecular analyses of a large series of liver tumor tissues confirm the association between OBI and HCC
2008—J Hepatol—Raimondo et al. [2]	Statements on OBI by an international, large panel of experts

**Fig. 1:**
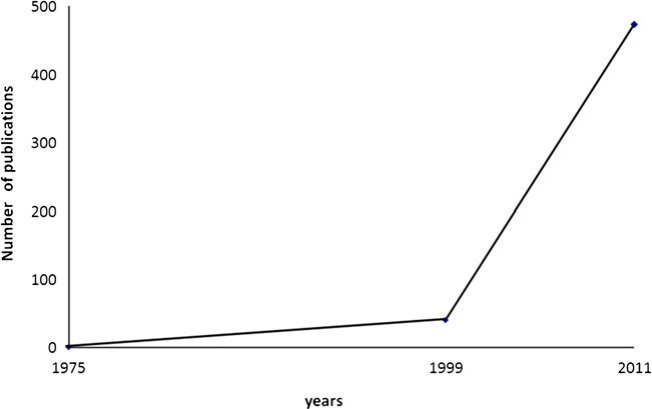
Publications on occult HBV infection over time

## Virology

HBV belongs to the Hepadnaviridae family, comprising hepatotropic DNA viruses able to infect mammalian and avian hosts and sharing with HBV most of the genetic structure and replicative characteristics [16]. HBV genome consists of a partially double-stranded relaxed circular DNA, approximately 3,200 nucleotides in length, and contains four partially overlapping open-reading frames (ORF), *pre-S/S*, *pre-C-C*, *P*, and *X*. *Pre-S/S* ORF, which encode the three viral surface proteins, preS1 (or Large), preS2 (or Middle), and S (or small), which correspond to HBsAg. *PreC/C* ORF encodes the core antigen (HBcAg) and the soluble antigen “e” (HBeAg). P ORF encodes the terminal protein (TP) and the viral polymerase that possesses DNA polymerase, reverse transcriptase, and RNaseH activities. X ORF encodes the regulatory X protein, which is essential for virus replication and is capable of transactivating the expression of numerous cellular and viral genes [17].

The replication cycle of HBV presents very particular characteristics that can be schematically summarized as follows [17]: (a) interaction of the virus with still unidentified cell surface receptors; (b) release of the core nucleocapsid into the cytoplasm and its transport to the nuclear membrane; (c) discharge of the HBV genome into the nucleus and its conversion into a covalently closed circular DNA (cccDNA); (d) transcription of cccDNA by the host RNA polymerase II into all viral mRNA, including a pregenomic RNA (pgRNA); (e) translocation of HBV transcripts into the cytoplasm, where their translation yields the viral envelope, core, “e”, polymerase, and X proteins; (f) assembly of nucleocapsids and, inside them, synthesis of new viral DNA from pgRNA by viral reverse transcriptase; (g) recycling of a small portion of nucleocapsids into the nucleus to maintain the reservoir of cccDNA stable; and (h) coating of most nucleocapsids with viral surface proteins in the endoplasmic reticulum and subsequent release of mature virions. HBV has been classified as a pararetrovirus because of some similarity with retroviruses. In fact, HBV—although a DNA virus—replicates through the reverse transcription of the pgRNA representing its intermediate replicative form. Similar to retroviruses, HBV DNA can integrate in the genome of the host hepatic cells but, unlike what happens for retroviruses, integration has no role in the replicative cycle of HBV and it involves only segments of the viral genome. Integrated HBV may persist forever in the liver cells of infected individuals even when they are HBsAg-negative. However, the presence of integrated viral DNA in HBsAg-negative subjects should not be strictly considered as occult infection, since this condition is essentially related to the intrahepatic long-lasting persistence of entire viral genomes as free episomal forms and, in particular, to the persistence of viral cccDNA, as a stable chromatinized episome, in the nucleus of the infected cells [18]. The stability and long-term persistence of viral cccDNA molecules together with the long half-life of hepatocytes imply that HBV infection, once it has occurred, may continue indefinitely over time [18, 19].

**Fig. 2:**
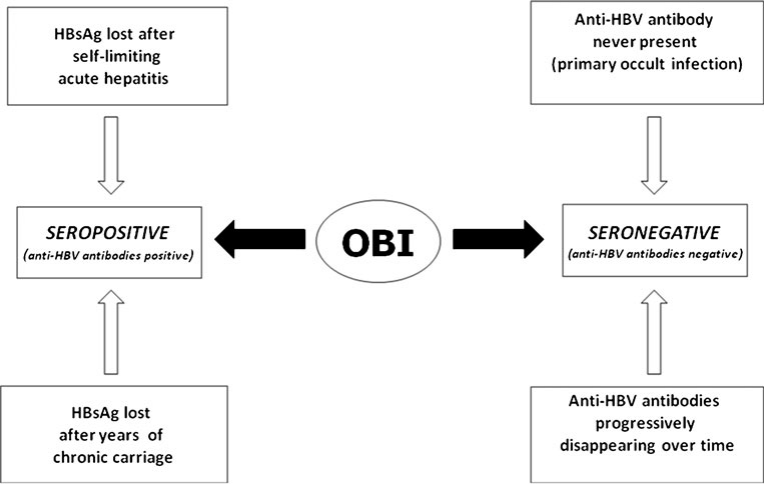
Schematic representation of the various conditions leading to different OBI serological profiles

The lack of detectable HBsAg in spite of the presence of episomal, free HBV genomes at intrahepatic level is attributable, in some cases, to the HBV genetic variability determining either infection with S gene variants (S-escape mutants) producing a modified HBsAg that is not recognized by commercially available detection kits (even when the most sensitive ones are used) or, in a small number of cases, infection with HBV mutants with defective replication activity or synthesis of S proteins [20]. However, in the majority of cases, “occult” HBV genomes are replication-competent viruses with grade and relevance of genetic heterogeneity comparable with the HBV isolates from individuals with “overt” (HBsAg-positive) infection [21]. Thus, it is generally believed that the OBI status is mostly consequent to a strong suppression of HBV replication and gene expression where different mechanisms can be implied. Before discussing these mechanisms, it is important to consider that although OBI is significantly associated with the presence of anti-HBV antibodies (namely, anti-HBc and anti-HBs antibodies directed against the viral core antigen and HBsAg, respectively), more than 20 % of occult-infected individuals are negative for all HBV serum markers [5]. Consequently, and according to the above-mentioned Taormina statements, one can distinguish seropositive (anti-HBc and/or anti-HBs positive) and seronegative OBI (anti-HBc and anti-HBs negative) individuals. In seropositive OBI, the HBsAg may become negative either quickly after the resolution of acute hepatitis or after years or decades of overt chronic HBV infection, whereas the seronegative OBI cases might have either progressively lost all HBV serum markers or might be HBV-negative since the beginning of the infection, similarly to what was observed in the woodchuck model of hepadnavirus infection with the woodchuck hepatitis virus (WHV) [22] (Fig. 2). Thus, OBI appears to shape up as a complex scenario with different virological and immunological profiles.

## Mechanisms potentially involved in HBV inhibition and OBI status induction

The experimental systems available at present are not suitable for an adequate study of HBV infection, which is a complex event characterized by different and often instable phases [1]. Thus, knowledge of the factors implicated in suppression of the viral activity up to the induction of occult HBV status is still very limited. Nevertheless, it would be of utmost importance to define these factors, since their elucidation would reveal important insights into HBV virology and provide information for new therapeutic strategies fitted. Below we will discuss data and hypotheses concerning host factors that are generally believed to be involved in the suppression of HBV replication and in control of the infection. Finally, we will also mention the possible role that viral factors and coinfection with other viral and nonviral agents may play in inducing OBI status.

### Host factors

Much evidence indicates that host factors are strongly implicated in the induction and maintenance of the occult status of HBV infection. Further proof is provided by a recent in vitro study showing that replication, transcription, and protein synthesis abilities of occult viral isolates can be fully restored once the viruses are taken out from the host’s liver microenvironment [21].

#### Immunological factors

Numerous clinical studies have clearly demonstrated since the 70s that all the conditions inducing immunosuppression (i.e., hematologic malignancies, chemo- or immunotherapies, etc.) may provoke the reactivation of OBI with the reappearance of the typical serological profile of overt active infection [4, 5, 20]. This is quite strong (though indirect) evidence of the involvement of the host’s immune surveillance in the OBI development. This involvement is also confirmed by the data showing that a long-lasting memory CD4 and CD8 cell responses against HBV antigens are still detectable several years after recovery from acute hepatitis B possibly because during the occult phase of the infection, HBV is still able to synthesize minute amounts of antigens, which are undetectable by available technical approaches but are sufficient enough to maintain an HBV-specific T cell response [8, 10]. Indeed, besides HBV cccDNA molecules, all viral transcripts have been detected in the liver of occult-infected individuals [9, 23, 24] and real-time PCR quantification has revealed small but still quantifiable amounts of intrahepatic HBV mRNA in these subjects [24]. Therefore, clinical recovery from HBV infection not only implies the lack of complete clearance of the virus but also reflects the ability of the immune system to keep under tight control leftover viruses in the liver after clinical resolution of disease.

In a recent study, Zerbini et al. demonstrated that OBI patients with and without anti-HBc antibodies displayed a different profile of HBV-specific T cell responses [25]. Indeed, although circulating HBV-specific T cells were detected in seronegative (anti-HBc negative) patients with occult infection at frequencies comparable with seropositive (anti-HBc positive) OBI subjects, in vitro expansion and IFN-γ production by HBV-specific T cells was much weaker than in OBI-seropositive individuals. On the basis of the evidence obtained in the woodchuck animal model, it has been hypothesized that these distinct behaviors of cell-mediated immune responses in seropositive and seronegative OBIs might reflect different modalities of HBV transmission [22]. Indeed, in the woodchuck model, exposure to low WHV doses (<10^3^ virions) may give rise to a persistent infection in the absence of viral serum markers. Interestingly, this so-called woodchuck “primary” occult infection does not confer protective immunity, suggesting that a functional memory T cell response is generated only after infection with a higher dose of inoculum [22].

A very recent study has largely confirmed the observations of Zerbini et al. by investigating HBV-specific immune responses in blood donors with OBI [26]. In these subjects, potent, multispecific HBV-specific T cell responses were observed. In particular, HBV-specific Th1 responses were quantitatively stronger in OBI than in inactive carriers and were similar or even higher against HBV antigens than those observed in patients with previous HBV resolution. These data led the authors to conclude that the host’s immune system has the ability to strongly suppress HBV replication, thus providing a plausible explanation to the very low (or even absent) viral loads and the lack of detectable HBsAg observed in OBI individuals [26].

Other relevant evidence on the importance of the host’s immune systems in OBI control derives from studies on HIV-infected patients [27]. It has been recently shown that in these patients HBV viremia, in cases of occult infection, is significantly associated with lower CD4 counts. In patients with CD4 counts >500 cells/mm^3^, no occult HBV was observed. Thus, cellular immune deficiency, as reflected by the reduced CD4 counts in HIV infection, might result in a decreased control of HBV activities, so that HBV can restart to replicate and become detectable.

Of interest, the study of cytokine expression in HIV-infected individuals has shown decreased levels of IL-8, IL-10, IP-10, sFas, and sFasL in OBI patients compared to patients with overt HBV infection [28]. In particular, sFas levels were significantly reduced during occult infection, suggesting a reduced inhibition of apoptosis in this condition which, in turn, could favor a partial viral clearance, thus contributing to OBI occurrence [28].

Several relevant data suggest that not only the adaptive but also the innate immune response may play a role in the control of viral replication. Experiments with HBV-replicating transgenic mice and chimpanzees have shown that inflammatory cytokines, such as interferon type 1 (IFN) and TNF-α, can efficiently suppress viral replication through noncytolytic, immune-mediated mechanisms [29]. Moreover, it was more recently demonstrated that liver cells can mount an effective innate response to HBV infection with the production of INF-β- and IFN1-stimulated genes, which, in turn, may control HBV replication [30]. Thus, one might speculate that innate immune response might be implicated in controlling HBV activities, particularly in seronegative OBI patients in whom poor in vitro T cell expansion has been observed.

#### Epigenetic factors

HBV cccDNA molecules accumulate in the nucleus of infected hepatocytes as stable minichromosomes packaged into nucleosomal arrays by histone and nonhistone proteins, similarly to host cell chromatin [18]. Nucleosomal organization of viral genomes has been described for several DNA viruses such as the polyoma viruses, herpesviruses, simian virus 40, and human and bovine papilloma viruses [31]. A number of recent discoveries have underscored the importance of chromatin dynamic changes in histone composition and modification in tthe regulation of gene expression and replication during the different stages of viral productive replication, latent infection, and reactivation from latency, heightening the possibility that epigenetic processes may dictate, at least in part, the outcome of infection [31]. Both Epstein–Barr virus (EBV) and herpes viruses (HHV), for example, make extensive use of epigenetic modifications of histones as a mechanism of transcriptional control during their latency status [32, 33].

We have recently developed a ChIP-based HBV cccDNA quantitative approach to study the recruitment in vivo of cellular and viral proteins onto the HBV minichromosome [34]. The HBV cccDNA ChIP assay couples a cccDNA ChIP technique with a sensitive and specific real-time PCR protocol for cccDNA quantification [18, 34, 35]. Using the cccDNA-ChIP assay, we were able to show that HBV genomes are targeted by epigenetic regulatory mechanisms. Firstly, we could confirm existing data concerning the recruitment, in vivo, of H3/H4 histones and of HBV core protein on the cccDNA minichromosome [34]. Subsequently, using the same approach, we and others showed that several cellular transcription factors, including CREB, ATF, STAT1, and STAT2, and different chromatin modifying enzymes can bind the cccDNA in cells replicating HBV [36, 37]. Indeed, using antiacetylated-H3 or antiacetylated-H4 cccDNA ChIP assay, we found that HBV replication is regulated, both in cell-based replication systems and in the liver of HBV chronically infected patients, by the acetylation status of H3/H4 histones bound to the viral cccDNA in the nuclei of HBV-infected hepatocytes. The corecruitment of histone acetyltransferases (PCAF and p300/CBP) paralleled viral replication in vitro, whereas histone deacetylase 1 (HDAC1) recruitment onto the cccDNA was correlated with low HBV replication in vitro and with low viremia in vivo [34]. The importance of cccDNA histone epigenetic modifications in the regulation of the viral transcription/replication is further confirmed by the observation that abrogation of PCAF expression by specific siRNAs reduces HBV replication and acetylation of cccDNA-bound histones, whereas the class I and class III histone deacetylase inhibitors trichostatin, valproate, and nicotinamide induce an evident increase of both cccDNA-bound acetylated H4 and HBV replication. Interestingly, valproate, which is used clinically as an anticonvulsant and in the treatment of bipolar syndromes, has been shown to reactivate lytic replication both of latent HHV8 and of EBV [38, 39]. As a note, a fatal reactivation of hepatitis B has been described in a 65-year-old Chinese woman who received radiation therapy with concurrent temozolomide because of a glioblastoma and valproic acid for seizure prophylaxis [40].

Preliminary data, obtained by applying the ChIP assay to liver tissues of patients with OBI, showed that in these subjects cccDNA-bound histones were hypoacetylated and the recruitment of chromatin-silencing factors as HDAC1, the heterochromatin proteins HP1, and the histone H3 K9 methylase SUV39H1 were increased. Thus, the profound suppression of transcription/replication of the occult HBV episome in OBI livers might occur at the level of chromatin organization (Pollicino et al, unpublished data).

Interestingly, recent evidence indicates that IFNα is able to inhibit cccDNA-driven transcription of genomic and subgenomic RNAs, both in HBV-replicating cells and in HBV-infected chimeric uPA/SCID mice repopulated with primary human hepatocytes. In particular, it was found that, in response to IFNα, cccDNA-bound histones become hypoacetylated and both components of the transcriptional repressor complex PRC2 are actively recruited on the cccDNA [41]. These observations support the hypothesis that the ability of IFNα to inhibit HBV replication may be mediated, also by an active epigenetic control of HBV minichromosome. In addition to post-translational modifications of histones, DNA methylation of CpG-rich regions (CpG islands) in the HBV DNA might also contribute to regulate HBV gene expression. [18, 42, 43].

### Viral factors

Since the lack of detectable HBsAg in serum is a characterizing feature of OBI, considerable interest has been focused on mutations in the surface gene and its regulatory regions. Thus, besides mutation clustering in key immunodominant regions of the surface protein able to decrease the immune recognition of the virus, deletions in the preS1 region impairing viral packaging, structural alteration in genomic regulatory regions leading to a strong reduction of HBsAg expression, and mutations affecting posttranslational production of HBV proteins have been described [2, 20, 44].

As stressed above, however, most of the studies have shown that these mutations are not detected in HBV isolates from the great majority of occult HBV-infected individuals and importantly, they are not unique to occult HBV but can be found in isolates from patients with overt HBV infections, including those with high viral loads [21, 26, 45, 46].

### Coinfection

HBV activity might be impaired by other infectious agents in cases with coinfection. In particular, HCV has been suspected to strongly suppress HBV replication up to the point where it determines OBI development in coinfected individuals. In fact, a number of in vitro studies had clearly demonstrated that the HCV “core” protein strongly inhibits HBV replication [47] and OBI shows the highest prevalence precisely in HCV-infected patients [5, 20]. However, more recent studies have brought into question the interplay between HCV and HBV, and when the in vitro cotransfection experiments were conducted with full-length HBV genomes and HCV replicon (thus, not limiting the study to a single HCV protein), no interference between the two viruses was noted [48]. Summarizing, the available data cannot allow definitive conclusion for any role of HCV in inducing OBI. Even individuals positive for human immunodeficiency virus (HIV) frequently show either overt or occult HBV coinfection, but there is no evidence of possible direct effects of HIV on HBV activity. Finally, other infectious agents might also potentially inhibit HBV and it has been shown that schistosoma mansoni is capable of strongly suppressing HBV replication in a transgenic mice model [49].

## Detection

Standardized, valid assays for occult HBV detection are not yet available. According to the above-mentioned Taormina statements, the gold standard for OBI testing is the analysis of DNA extracts from liver tissues performed by the use of highly sensitive and specific techniques, i.e., nested polymerase chain reaction (PCR) or real-time PCR, and of oligonucleotide primers specific for different HBV genomic regions and complementary to highly conserved (genotype shared) nucleotide sequences [2]. However, liver tissue specimens are available only in a minority of the cases, since needle liver biopsy cannot be performed in most subjects. Thus, the analysis of serum or plasma samples is the most common approach to identify occult HBV-infected cases. In this context, to improve the sensitivity of the test performing DNA extraction from at least 1 ml of serum (or plasma) and serially testing collected samples has been suggested in consideration of the fluctuating profile that detectable viremia often presents in OBI patients [2]. If highly sensitive HBV DNA testing is not feasible, the serological assay for anti-HBc antibody should be used as a possible surrogate marker for identifying potential seropositive OBI individuals in cases of blood, tissue, or organ donation and when immune suppressive therapy has to be used. In this context, it must be stressed that not all anti-HBc positive individuals are found to be HBV DNA-positive and that anti-HBc tests may provide false positive results [2, 50, 51].

## Prevalence

Considering the HBV life cycle with a long-lasting persistence of viral genomes in most infected patients independently of HBsAg status, OBI is expected to be a worldwide diffused entity, which prevalence is higher in populations at high risk of parenterally transmitted infections and generally dependent on the level of HBV endemicity in the different geographic areas. In addition, there is considerable evidence indicating that OBI is highly prevalent also in individuals with CLD and, in particular, in HCV chronically infected patients [5, 20]. In fact, HBV DNA is detectable in about one-third of HBsAg-negative HCV carriers in the Mediterranean Basin and in more than 50 % in Far East Asian countries [5]. Of note, recent studies conducted in the USA on patients of Caucasian origin undergoing liver transplantation for HCV-related cirrhosis showed that 50 % of these individuals were OBI-positive [52]. These data are particularly surprising and relevant taking into account that HBV prevalence in the Caucasian American population is one of the lowest in the world [53]. OBI has been less investigated in patients with HCV-negative CLD. Its prevalence has been reported to range between 20 % and 30 % in subjects with cryptogenic liver disease [5, 20, 23]. In one study, 12.2 % of patients with chronic hepatitis related to autoimmune disorders proved OBI-positive when serum samples were tested, although this prevalence appeared to significantly increase when viral DNA was also assayed on liver extracts of a number of those patients [54].

Populations at high risk of parenterally transmitted infections have been widely investigated for occult HBV. A high prevalence has been reported in intravenous drug addicts in Baltimore (45 %) [55] and in hemophiliacs in Japan (51 %) [56]. Studies on hemodialysis patients have provided widely divergent results that cannot be explained solely with the level of HBV endemicity in different areas [20]. In fact, even some studies carried out within the same country reported contradictory data [57–60], thus suggesting that the differences in sensitivity and/or specificity of the methods used in the various studies could be the main cause responsible for the discrepant findings. Of course, since liver biopsy cannot be routinely performed in hemodialysis patients, no study evaluating this setting of patients carried out the analysis of occult HBV on liver specimens. Furthermore, no study longitudinally evaluated the presence of viral DNA in serially collected serum samples. Thus, we might suppose that OBI prevalence is generally underestimated in this subset of patients. Even more evident is the divergence among the numerous reports concerning OBI prevalence in HIV-infected patients [20]. Of note, the highest prevalence was found when the most sensitive techniques for OBI detection were used and when longitudinally collected multiple patient sera were tested. However, only one study evaluated the presence of HBV sequences at intrahepatic level, showing that 41 % of HIV/HCV coinfected Italian patients also carried OBI [61]. OBI has been extensively explored in blood donors where it appears to occur quite rarely in the western world, whereas it is frequently detected in developing countries [20]. On the contrary, OBI has been much less investigated in the general population so far. In a study evaluating HBsAg-negative residents of a Canadian Inuit community, HBV DNA was detected in 18 % of anti-HBc positive subjects and in 8 % of HBV seronegative individuals, respectively [62], whereas occult HBV genomes were found in 16 % of Korean HBV/HCV-negative healthy subjects with normal transaminase values and in 15.3 % of healthy hematopoietic stem cell donors from Hong Kong [63, 64]. We recently investigated the presence of HBV DNA at intrahepatic level in 98 liver disease-free HBsAg-negative Italian individuals who underwent liver resection or needle liver biopsy during abdominal surgery: 16 of them (16 %) were OBI positive, most having circulating anti-HBV antibodies [65].

## Clinical implication

The great interest in OBI is mainly related to the growing, widely debated evidence of its clinical impact. Actually, OBI may be involved in several different clinical contexts that we schematically group as follows: transmission of the “occult” virus (mainly through blood transfusion and orthotopic liver transplantation, OLT) with consequent hepatitis B in the recipient; reactivation of the infection and consequent (re)development of the HBV-related liver disease; effect on occurrence and progression of the CLD; and role in hepatocarcinogenesis. Here, we schematically discuss these different clinical contexts of OBI involvement and cite recent reports which have suggested a possible involvement of OBI also in malignancies other than HCC.

### Risk of OBI transmission

#### Blood transfusion

It is well established that carriers of occult infection may be a source of HBV transmission in the case of blood transfusion with the consequent development of a typical type B hepatitis in the recipients. Recently, this aspect has been the focus of several extensive reviews and articles and we refer to them for an exhaustive discussion of the theme [50, 51, 66–68]. Here, our scope is to stress some particular aspects that have relevance in the general debate on OBI.

In the last 20 years, the risk of HBV infection after blood transfusion has dramatically decreased due to the implementation of progressively more sensitive and specific diagnostic tests. In fact, post-transfusional hepatitis B is, at present, a rare event in the western world, although some residual cases still occur [69]. Schematically, three conditions may be responsible for the transfusional transmission of HBV:

1. The donor is in the *window period* (the HBsAg-negative, viremic, early acute phase of HBV infection). This is not the subject of the present paper and, in any case, it accounts for a minority of OBI-positive blood donors [68].
2. The donor is a typical “OBI carrier” with a wild-type virus which replication activity and gene expression are suppressed. This point is important and intriguing, and it should be taken into account that OBI infection is characterized by periods of transient HBV viremia alternating with periods in which the viral DNA is undetectable in the serum [50, 70, 71]. Thus, an occult HBV-infected individual may have a profile of blood infectivity fluctuating over time. However, one important point is the real ability of the very low amount of serum HBV DNA usually present in OBI carriers to generate acute hepatitis B in the recipient. It seems that HBV DNA-positive donors with anti-HBc as the only serological marker are more infectious than those carrying anti-HBs [68]. In an animal model of chimpanzees, the minimum 50 % infectious dose (CID50) of HBV was estimated to be approximately ten copies [72], whereas in humans, it is far from being established [68], although the possibility of inducing acute hepatitis is likely dependent on the viral load, the amount of plasma transfused, the immunocompetence of the recipient, and the HBV serological status (presence/absence of anti-HBc and/or anti-HBs) in both donor and recipient. Nevertheless, it should be considered that the lack of acute hepatitis development does not exclude HBV transmission and infection of the recipient who, in turn, might theoretically become occult HBV-infected.
3. The donor is infected with variant HBV strains (S-escape mutants) that are replication-competent but produce abnormal surface proteins that are not recognized by the commercially available HBsAg detection kits. This condition appears to be a major cause of the residual cases of HBV transmission by blood transfusion. In this context, the use of multivalent anti-HBs antibodies in the HBsAg detection kits has been recently recommended by an expert panel for the identification of blood donors infected by HBsAg escape mutants [2], even if this strategy does not definitely solve the problem of post-transfusion HBV infection, especially in geographic areas where HBV infection is highly endemic and the viral genomic variability is potentially the highest.

The introduction of Nucleic Acid Testing (NAT) for HBV was intended to identify all blood donors with circulating HBV DNA independently of each of the above reported conditions. Actually, NAT for HBV has revealed that a small part of HBsAg-negative blood donors worldwide have detectable amount of HBV DNA in the serum. Although data are not homogeneous due to the different population examined (i.e., first or repeated blood donors, replacement blood donors, or general population) and to the different sensitivity of the assays used, what clearly emerged in the studies based on the NAT technique is that the frequency of HBV DNA detection in HBsAg-negative subjects varies considerably according to the prevalence of the infection in the different geographical areas. In addition, these studies have shown that OBI and HBV S-escape mutant infections may be identified in anti-HBc-positive samples (approximately 50 % of which also carrying anti-HBs) but also in rare cases of anti-HBs without the presence of anti-HBc, as has been described in vaccinated and nonvaccinated blood donors [66, 73].

#### Organ transplantation

The HBV transmission from an OBI donor in the event of OLT is a well known and frequent cause of de novo hepatitis B in cases where the recipient is HBV naïve [20, 74]. This is the obvious consequence of the fact that the hepatocytes are the reservoir of HBV cccDNA (indeed occult HBV appears to have very low rates of occurrence in cases of kidney, heart, and bone marrow transplantation [20]) and it is the basis for the general agreement in performing anti-HBV prophylaxis (with hepatitis B immunoglobulin, lamivudine, or their combination) in HBsAg-negative transplanted patients who receive livers from anti-HBc positive donors (of note, OBI transmission from HBV seronegative individuals is uncertain and remains difficult to recognize). This prophylaxis appears to be very effective in preventing de novo HBV hepatitis in the recipients [75] but not to avoid HBV reinfection [74]. In fact, several recent studies have clearly demonstrated the frequent occurrence of OBI in transplanted individuals who were occult-infected prior to OLT and/or received the new organ from an OBI carrier. In the “new” liver, HBV genomic sequences (and also HBV cccDNA) are present and may derive from occult viruses previously infecting the recipient, the donor, or even both [76]. Whether OBI in these conditions might have any clinical relevance in long-term outcome has to be clarified, although some preliminary evidence suggests a possible involvement in faster progression of post-transplant liver disease in HCV-positive patients [77]. Of note, OBI also develops in HBsAg-positive transplanted patients who are treated with hepatitis B immunoglobulins and lamivudine and become HBsAg-negative in the post-OLT period [76].

### OBI reactivation

The possible HBV reactivation in patients with diseases involving the immune system or undergoing immuno- and/or chemotherapy is a well-known phenomenon. This is a field of growing interest also as a consequence of the availability of new, potent immunological drugs used in different clinical contexts. HBV reactivates very frequently in HBsAg-positive individuals undergoing immunosuppression, and this event may occur also in OBI patients where it is often associated with the fulminant course of hepatitis [78]. Consequently, HBV reactivation in immunocompromised patients has recently been the focus not only of many studies but also of meetings, reviews, and position papers [79–81]. Moreover, it is an argument subject dealt with in all international guidelines for the management of HBV infection published in the last few years. Here, we briefly and schematically report the aspects we consider preeminent in the field of viral reactivation in OBI patients.

Thus, the strong suppression of viral replication and gene expression typical of the occult HBV status may be discontinued in patients under conditions of immunosuppression, who may consequently have a reactivation of the viral replication because of the drop in immunological control. As mentioned above, this is very important, though indirect, proof of the role played by immune control in inducing OBI. Of note, however, recent reports indicate that also the use of histone deacetylase inhibitors may be associated with OBI reactivation [40, 82], confirming the involvement of epigenetic mechanisms in the control of HBV activities and, consequently, modifications of viral cccDNA minichromosome structure and dynamics as possible causes of viral reactivation.

One main question is how frequent the viral reactivation in OBI patients is. Surely, it occurs more rarely than in HBsAg-positive cases, and it likely varies depending on different clinical settings and therapeutic treatments (Table 2), hematological malignancies, hematopoietic stem cell transplantation, and treatments including rituximab conditions being at high risk [20, 83, 84]. In this context, we would like to stress that OBI reactivation is usually diagnosed when it is followed by the occurrence of acute hepatitis. However, there is clear evidence that OBI individuals may frequently change their HBV serological profile if immunocompromised: in fact, anti-HBs positive individuals may lose this antibody during immunosuppressive therapy and two distinct studies have revealed that HBsAg re-seroconversion often occurs in subjects undergoing hematopoietic stem cell transplantation, although only a minority of these cases develop clinically typical acute hepatitis [85, 86]. Consequently, one might speculate that OBI reactivation is a quite frequent occurrence in itself, but it is rarely followed by a clinically acute event, thus its recognition and diagnosis might be missed in many cases.

**Table 2:** Conditions reported to be associated with OBI reactivation

Clinical conditions	Therapies
Hematological malignancies	ABVD
Non-Hodgkin lymphoma	BEAM
Hodgkin lymphoma	CHOP
Multiple myeloma	R-CHOP
Myelo-monoblastic acute leukemia	R-FND
Chronic lymphocytic leukemia	VAD
Hematopoietic stem cell transplantation	Temozolomide
Liver transplantation	Rituximab
Bone marrow transplantation	Adalimumab
Kidney transplantation	Tocilizumab
HIV infection	Abatacept
Rheumathoid arthritis	Infliximab
Glioblastoma	Etanercept
	Corticosteroids
	Methotrexate
	Leflunomide
	Bucillamin
	Valproic acid
	Romidepsin


*ABVD* adriamycin, bleomycin, vinblastine, dacarbazine; *BEAM* carmustine, etoposide, cytarabine, melphalan; *CHOP* cyclophosphamide, adriamycin/doxorubicin, vincristine, prednisone; *R-CHOP* rituximab and CHOP; *R-FND* rituximab, fludarabine, mitoxantrone, dexamethasone; *VAD* vincristine, adriamycine, dexamethasone

While prophylactic anti-HBV therapy with nucleos(t)ide analogues (NA) is a well-consolidated practice for the prevention of reactivation in HBsAg-positive patients undergoing immunosuppressive therapies, the prophylactic antiviral treatment in cases of patients suspected to be OBI-positive (HBsAg-negative, anti-HBc +/− anti-HB-positive) is still an argument of debate. The guidelines for the management of CHB licensed by the EASL in 2009 recommended testing these patients for HBV DNA and treating them as HBsAg-positive subjects when viral DNA is detectable, whereas when it is undetectable, they should be followed carefully by means of ALT and HBV DNA testing and treated with NA therapy upon confirmation of HBV reactivation before ALT elevation [1]. The scope of this narrow surveillance is clearly to recognize the viral reactivation in a phase anteceding the beginning of liver injury to prevent hepatitis development. In fact, once this event is established, the NA treatment is not always effective probably due to the late start of the antiviral therapy. Of note, some recent reports indicate that the use of the most potent NA drugs may still make it possible to efficaciously cure acute, severe hepatitis following OBI reactivation [87, 88].

### Occult HBV infection and chronic liver disease

A major and largely debated topic is whether occult HBV may provoke (or contribute to) liver damage and in which cases (or conditions) this event may occur or may be relevant from a clinical point of view. In this context, it seems important to consider that although individuals who recover from self-limited acute hepatitis persistently carry HBV genomes over time without showing any clinical or biochemical sign of liver damage [20, 65] when the hepatic tissue of such subjects was examined, histological patterns of a mild necroinflammation were detected up to several decades after the resolution of the acute hepatitis [89, 90]. These observations are in accordance with studies on the woodchuck model showing that animals convalescing from acute WHV hepatitis show lifelong persistence of small amounts of replicating virus associated with mild liver necroinflammation continuing for life [22]. Much evidence indicates that OBI is associated with the most severe forms of liver disease in HCV-infected patients, thus suggesting that occult HBV might favor or accelerate the progression of HCV-related CLD [5, 20]. Of note, recent reports have shown an association between phases of a rise in ALT levels and reappearance of circulating HBV DNA in patients with chronic hepatitis C and combined OBI, thus suggesting an active role of transient reactivation of HBV replication in liver cell injury [70, 71]. Altogether these data seem to confirm our previously reported hypothesis that, at least in conditions of immune competence, the occult infection is inoffensive in itself, but when other important causes of liver damage coexist (i.e., HCV infection, alcohol abuse, etc.), the minimal lesions produced by the immune response to the occult virus might contribute to making the course of the liver disease worse over time [91].

As a note, it is correct to mention that several clinical studies performed in the 1990s suggested that OBI may negatively influence the response to IFN therapy in chronic hepatitis C patients [5, 20]. Although the hypothesis that OBI may *help* HCV to resist IFN is intriguing, it has to be pointed out that all these studies concerned treatment schedules using conventional IFN therapy, whereas whether occult HBV may interfere with the response to pegylated-IFN plus ribavirin has not been adequately investigated so far. In any case, one may imagine that the advent of direct antiviral agents for the cure of HCV will likely make this topic flimsy from a practical point of view.

On the basis of the above-mentioned hypothesis that occult HBV might be unable to produce severe hepatic injury by itself, it appears difficult to provide an explanation to the considerable evidence indicating that OBI is associated with the progression of liver fibrosis and cirrhosis development also in patients with cryptogenic liver disease [5, 20]. Plausibly, a portion of these cases concerns individuals with a previously productive HBV infection where a progressive reduction of viral replication and serum HBsAg amount occurs. The HBsAg may even disappear over time despite the presence of severe liver disease that had been provoked by the overt B hepatitis and then be maintained once the occult HBV status develops. Of course, one cannot exclude that unrecognized causes of hepatic injury might concur with OBI to the induction of other cases of cryptogenic CLD.

### Occult HBV infection and HCC

A large body of evidence shows that HBV infection is the main risk factor for HCC development and the World Health Organization includes HBV in “group 1” human carcinogens, classifying it among the most important oncogenic agents after tobacco smoking [16]. Furthermore, many epidemiological and molecular studies performed since the 80s indicate almost unanimously that HBV persistence may play a critical role in the development of HCC also when the occult status of the infection occurs [16, 92]. This evidence is validated by a recent meta-analysis evaluating the studies focusing on this topic and confirming that OBI is an important risk factor for HCC development both in HCV-infected and HCV-negative patients with CLD [93]. Among HCV-negative patients, OBI appears able to exert its pro-oncogenic role in individuals without known causes of liver damage as well as in alcoholics and individuals with virus unrelated liver pathologies [20, 23, 94]. In this context, of great importance is a recent population-based cohort study conducted for more than two decades on Taiwanese mothers screened for HBV infection at each delivery and demonstrating that the risk of HCC development was significantly higher in women with persistent HBsAg-positive status, but among the HBsAg-negative mothers, those who underwent HBsAg sero-clearance during follow-up had a significantly higher risk of HCC development compared to HBV-unexposed women. In fact, this study indicates that HBV maintains its pro-oncogenic role also in the occult status and even in women that are known to be much less prone to develop liver cancer than men [95]. Finally, experiments in animal models showed that both woodchucks and ground squirrels, once infected by the corresponding hepadnaviruses, are at high risk of developing HCC also after the apparent clearance of the virus with disappearance of the viral surface antigen and seroconversion to the corresponding antibody [16].

Complex and multifactorial pathogenetic mechanisms underlie HCC development and much evidence reveals that HBV is involved in many of these mechanisms. In particular, chronic HBV infection is believed to exert its pro-oncogenic properties through both indirect and direct mechanisms: the indirect mechanisms are related to its propensity to induce continuous or recurrent phases of liver necroinflammation and to promote the progression of chronic hepatitis to cirrhosis (which is the step preceding the development of HCC in the vast majority of the cases); the direct carcinogenic mechanisms have been related to the ability of HBV to integrate into the host’s genome and to produce proteins, mainly X protein and truncated preS-S protein, provided with potential transforming properties. Considering that in OBI status (a) HBV DNA can persist in the hepatocytes both integrated into the host genome and as free episome; (b) the virus maintains its replication and transcriptional activity and ability to synthesize proteins, albeit at very low levels; and (c) the occult virus may determine a mild but continuous status of chronic necroinflammation and might contribute to progression toward cirrhosis, it is generally believed that OBI can contribute to hepatocellular transformation through the same direct and indirect mechanisms that subtend HCC development in overt HBV infection.

### Other tumors where OBI involvement has been hypothesized

Recent data indicate an association between HBV infection and malignancies other than HCC, such as intrahepatic cholangiocarcinoma and non-Hodgkin lymphoma [96–98]. Some evidence also suggests a possible involvement of OBI in these conditions [99, 100]. The tumorigenic mechanisms exerted by HBV in both these conditions need be totally elucidated, although the origin from the same progenitor cells of hepatocytes and cholangiocytes, on one hand, and the supposed lymphotropism of HBV, on the other hand, provide some theoretical basis for this intriguing topic. Of course, large cohort studies must be performed to verify these data firstly in HBV infection and, subsequently, in cases of OBI.

## Summary and conclusions

The availability of highly sensitive molecular biology techniques made it possible to disclose several virological aspects of OBI, to show its worldwide diffusion, and to reveal its possible implication in various clinical contexts. It appears well established that the molecular basis of OBI is related to the long-term persistence of HBV cccDNA in the nuclei of the hepatocytes, and OBI can be considered as a phase in the natural history of chronic HBV infection. The mechanisms determining OBI status have still to be mostly elucidated, but it is evident that viral factors (i.e., HBV genetic heterogeneity) have no relevant role in the majority of cases, while host factors appear to play an essential role by suppressing the viral activities. These biological aspects have clinical repercussions, since environmental changes leading to the breakdown of the host–virus balance may determine the reactivation of the virus replication ability and the development of a “typical” hepatitis B. This event may occur when the immunological status of an OBI patient is modified as in cases undergoing immunosuppressive therapies but also when drugs able to interfere with the epigenetic control of the HBV replication are used. In analogy, because of their replication competence, when occult viruses are transmitted to other individuals, they may induce hepatitis B. In fact, OBI is responsible for the large majority of the residual cases of transfusion-transmitted HBV hepatitis that is a rare event in the most developed countries but is still a quite serious problem in areas where the high HBV endemicity and the elevated costs of NAT diagnostic approaches hamper a totally efficient check of all transfusion blood units. HBV may be also transmitted in cases of OLT from an OBI-positive donor. In these cases, the event of de novo hepatitis B is, at present, prevented by the use of anti-HBV prophylaxis in the recipient. A widely debated aspect concerns the possible involvement of OBI in the progression toward cirrhosis of patients with chronic hepatitis. In this context, one should consider that OBI is a complex entity which includes several clinical/virological conditions quite different from one another such as, (a) patients rapidly recovered from self-limited hepatitis, (b) patients who had overt CHB for years or decades before losing the HBsAg, and (c) HBV seronegative patients, most of whom had likely been infected with minute amounts of viruses insufficient to induce a strong, specific immune response. One cannot rule out the possibility that these conditions may have a different impact on the outcome of the liver disease, and studies aiming at investigating this important feature must be performed in the near future. Finally, OBI appears to maintain most of the pro-oncogenic properties usually attributed to the overt HBV infection and quite solid evidence indicates that it is a risk factor for HCC development. However, several aspects still need to be elucidated, such as the epidemiology of OBI-associated HCCs. Moreover, molecular studies addressed at analyzing liver samples from large series of OBI-associated HCC compared to both overt HBV-associated HCC and HBV-negative HCC are still lacking. OBI is a fascinating field of viral hepatitis research and learning about it is fundamental for an overall understanding of HBV infection. One main issue that OBI studies have contributed to provide is that HBV, once infection occurs, is not eradicable even by the use of the most potent anti-HBV drugs at present available. Thus, the only chance to really win the war against this terrible (and “smart”) enemy of human health is to extend neonatal vaccination to all the countries, particularly where the virus is endemic.
